# Environmental heterogeneity blurs the signature of dispersal syndromes on spatial patterns of woody species in a moist tropical forest

**DOI:** 10.1371/journal.pone.0192341

**Published:** 2018-02-16

**Authors:** Pablo Ramón, Eduardo Velázquez, Adrián Escudero, Marcelino de la Cruz

**Affiliations:** 1 Departamento de Ciencias Biológicas, Universidad Técnica Particular de Loja, San Cayetano Alto, Loja, Ecuador; 2 Universidad de Magallanes, Centro Universitario de Coyhaique, Coyhaique, Chile; 3 Área de Biodiversidad y Conservación, Departamento de Biología y Geología, ESCET, Universidad Rey Juan Carlos, Madrid, Spain; Chinese Academy of Forestry, CHINA

## Abstract

We assessed the relative importance of dispersal limitation, environmental heterogeneity and their joint effects as determinants of the spatial patterns of 229 species in the moist tropical forest of Barro Colorado Island (Panama). We differentiated five types of species according to their dispersal syndrome; autochorous, anemochorous, and zoochorous species with small, medium-size and large fruits. We characterized the spatial patterns of each species and we checked whether they were best fitted by Inhomogeneous Poisson (IPP), Homogeneous Poisson cluster (HPCP) and Inhomogeneous Poisson cluster processes (IPCP) by means of the Akaike Information Criterion. We also assessed the influence of species’ dispersal mode in the average cluster size. We found that 63% of the species were best fitted by IPCP regardless of their dispersal syndrome, although anemochorous species were best described by HPCP. Our results indicate that spatial patterns of tree species in this forest cannot be explained only by dispersal limitation, but by the joint effects of dispersal limitation and environmental heterogeneity. The absence of relationships between dispersal mode and degree of clustering suggests that several processes modify the original spatial pattern generated by seed dispersal. These findings emphasize the importance of fitting point process models with a different biological meaning when studying the main determinants of spatial structure in plant communities.

## Introduction

Ecologists have widely accepted that most of the mechanisms acting in plant populations and communities leave an imprint in their spatial structures [1,2]. As a corollary, by studying the spatial patterns of plant populations and communities it is possible to infer which are their major underlying processes [3–5]. Seed dispersal has been recognized as a key factor to explain spatial structures of tropical forests [6,7] because it is critical for the recruitment of new individuals [7,8] and also for the regeneration of plant populations and communities [9]. In these megadiverse forests the spatial pattern of the majority of woody plant species show aggregation [10–12]. These clumped patterns have been easily explained because most seeds recruit near the parent trees, where the seed rain is always more intense [13–15]. This *dispersal limitation* to remain in the vicinity of mother trees should play a major role in tropical forests not only to determine conspecific spatial patterns, but to promote species coexistence by inducing heterospecific segregation and limiting competitive exclusion of the poorest competitors [16–18]. Seed dispersal lays the spatial template from which plant populations start to develop. If true, these first spatial arrangements could be blurred or exacerbated by subsequent life-history processes before reaching the adult stage [7,8]. In this sense, environmental heterogeneity plays an important role in seedling establishment and sapling survival [6], thereby influencing conspecific spatial patterns in many tropical forests (e.g. [19,20]) an accentuating the primary pattern. Thus spatial aggregation induced by dispersal limitation could be reinforced by species-specific habitat associations at relatively small scales [21,22], but it can be also disrupted by density-dependent mortality due to the attack of natural enemies such as insects or pathogens [23,24]. In any case, there is an ample consensus that dispersal limitation and environmental heterogeneity are the two most critical factors in determining the spatial realized patterns of mature woody plant species in tropical forests [25–28]. Disentangling the relative importance of these two processes as determinants of the spatial aggregation of most species is crucial to decode mechanisms of species coexistence in megadiverse communities [3,29], but it is still far from clear [27]. Moreover, it has been traditionally assumed that clumping at local (plot) scales is determined almost exclusively by dispersal limitation [12,30], whereas the effects of environmental heterogeneity occur at larger (landscape) scales [31]. Recent evidence, however, suggests effects of these two processes can overlap at fine spatial scales [28,32,33]. Note that in some rare cases, environmental heterogeneity and dispersal limitation could affect spatial patterns at exactly the same scales and this would complicate the selection of the appropriate model. Spatial point process models, which are able to generate the expected pattern of a plant population according to *a-priori* selected mechanistic hypotheses [34], are increasingly used to describe stem-maps and quantify their governing processes [5,35]. A set of candidate models can be simultaneously tested and compared with realized patterns to discern what mechanisms could produce a spatial signal compatible with observations. Among them, inhomogeneous point processes (hereafter, IPP) have been used to describe spatial patterns of particular species which are driven almost entirely by environmental heterogeneity, such as those species confined along water courses [12] or in edaphically heterogeneous sites [36]. Homogeneous Poisson Cluster Processes (hereafter, HPCP), which simulate the dispersal of a number of offspring individuals from a random initial arrangement of parent trees, adequately describe conspecific spatial patterns in Neotropical [11] and South East Asian [10,12] tropical forests. Additionally, Inhomogeneous Poisson Cluster Processes (hereafter, IPCP) are able to simulate the joint effects of dispersal limitation and environmental heterogeneity [37].

Seed release phenology, ripening timing and propagule morphology are key life traits for plant regeneration [38] which are evolutionary controlled and tightly related to the dispersal syndrome [39]. As a consequence it could be expected the critical intervention of these syndromes in the configuration of realized distribution patterns in these megadiverse forests. For instance autochorous tree species (i.e. those dispersed by gravity, gyration or ballistic means) should have the smallest cluster sizes as a consequence of their limited dispersal. Anemochorous (i.e. wind-dispersed) species frequently should exhibit tighter clusters than zoochorous (i.e. animal-dispersed) ones because their dispersal distances are negatively affected by the intricate structure of the forest canopy and its influence on wind speeds [40,41]. Among zoochorous species, it could be hypothesized that those producing small fruits had tighter clusters than those that producing larger fruits, because the latter are eaten by large birds and terrestrial mammals with large home ranges that move seeds over longer distances than small birds and mammals [42]. However, this picture is not definitive since earlier studies showed that large frugivores usually defecate masses of seeds and may cause aggregated spatial patterns, whereas small frugivores such as birds and bats deposit single seeds, resulting in isolated recruits and therefore, resulting in more scattered conspecific patterns [43,44]. More recent evidence in Barro Colorado Island -BCI- [45] and in an Ecuadorian dry forest [28] provides support for this last hypothesis. Together these studies suggest that, although species with different dispersal syndromes will have different cluster sizes, the strong relationships found by [30] in the BCI forest between dispersal syndromes and cluster size might be not so general. Therefore, whether the initial spatial patterns are maintained after subsequent life stages, or are blurred by environmental heterogeneity, remains as a relevant issue required to explain coexistence of species in megadiverse communities.

In this study we aim to assess whether conspecific spatial patterns associated to different dispersal syndromes are modified by the effects of environmental heterogeneity. To this end, we re-analyze the spatial patterns of 229 woody plant species in the moist tropical forest of BCI (Panama) previously used by [30]. We checked if these patterns were best fitted and able to explain observed patterns by IPP, HPCP and IPCP, which describe the tree distributions generated by environmental heterogeneity, dispersal limitation, and their joint effects, respectively. In particular, we sought to answer the following questions: (1) is there evidence of heterogeneity in the spatial patterns of the different species? And (2), does abiotic heterogeneity affect the spatial signatures of the different dispersal syndromes? Following the premise that abiotic heterogeneity may affect the survival of newly recruited individuals by sieving the pattern originated from seed dispersal, we considered the following hypotheses: (i) conspecific spatial patterns of the vast majority of species will be faithfully reproduced by IPCP, because the first template for tree distribution is subject to the effects of local environments; (ii) spatial patterns of anemochorous species will be best fitted by HPCP, reflecting that anemochory is quite a limited dispersal strategy in BCI producing small clusters that would not be affected by coarse scale heterogeneity; and (iii), the relationships between cluster sizes and dispersal syndromes of the species will be less strong and significant that those reported by [30]. Finally the point processes that best describe the spatial pattern of the species will yield (iv) the smallest cluster sizes for autochorous species, (v) smaller cluster sizes for anemochorous than for zoochorous species.

## Materials and methods

### Study area

Our study was carried out in the 50 ha Barro Colorado Island Forest Dynamics Plot (BCI), Panama (9° 9’ N; 79° 51’ W). This plot comprises an altitudinal range of 120–155 m.a.s.l. and has a gentle topography, with slopes of 7–20°. Rainfall averages 2600 mm•yr^-1^, falling mainly between April and November. The BCI plot contains a semi-deciduous moist tropical forest in which all woody plant individuals ≥ 1 cm DBH (diameter at 1.3 m height above ground) were mapped, measured and identified to species in 1982 in the first instance, and every 5 years since 1985 [46,47]. Early in the first census, [48] observed that in this forest the majority of species showed clumped patterns. However, these patterns are not necessarily associated with topographic features [49], but with underlying soil chemical properties [20]. In this plot, where there is a strong seed limitation [13], primary dispersal of 73% of the woody plant species is by animals, whereas that of 26% is by wind, water and ballistic means [40].

### Data collection

Following [30], we used the 1995 census data, which includes 229 woody plant species represented by at least 20 individuals within the plot. We did not consider species with fewer individuals also because the accuracy of spatial pattern modelling relies on reasonable minimum population sizes. We classified these species according to their dispersal syndromes; autochorous (i.e., species dispersed by ballistic or gyration means), anemochorous and zoochorous (i.e., species dispersed by wind and animals, respectively) [40]. Among zoochorous species we also classified according to small fruit-size (< 2 cm diameter, n = 115 species), medium fruit-size (2–5 cm diameter, n = 57) and large fruit-size (> 5 cm diameter, n = 28).

### Point process models and statistical analyses

To characterize the spatial pattern of each species we used Ripley’s K function [50]. For a homogeneous point pattern where *λ* is pattern intensity (also termed *point density*), *λK*(*r*) is the expected number of points (conspecific individuals) within a circle of radius *r* around an arbitrary point (i.e. a focal tree). If individuals are randomly distributed, then the expected value of *K*(*r*) is *πr*^*2*^. For each species, we also calculated the summary statistic *K*(*r*)/ *πr*^*2*^, at intervals of 10 m, from *r* = 0–250 m. This statistic is > 1 when the spatial pattern of a particular species is more aggregated than random at distance *r*, and is < 1 when it is more regular than random [30]. Mathematical details are provided in supplementary material (S1 Text).

We compared the (observed) pattern of each species, characterized by *K*(*r*), with the (expected) pattern generated by three different spatial point processes which model the spatial point patterns generated by environmental heterogeneity, dispersal limitation and the joint effects of these two factors [28,32]. To account for the effect of environmental heterogeneity, we used an IPP, also termed heterogeneous Poisson process [51], which assumes that the spatial location of points (i.e. woody plant individuals) is independent from each other, but pattern intensity varies throughout the study area. It is assumed that these variations are caused by environmental heterogeneity [26]. To examine (and model) the effects of dispersal limitation we used a HPCP. This process generates an aggregated pattern in a two-steps procedure: first, it creates a point pattern of cluster centers with constant intensity and, subsequently, each parent produces a number of off-spring individuals which are dispersed around the parents according to a radially symmetric Gaussian distribution with mean zero and standard deviation *σ* [34]. As *σ* is the standard deviation of the distance between each offspring and its parent points, the average cluster size of the point pattern yields ~ 2*σ* [35]. Therefore, this parameter can be used as a valid estimation of the average cluster size [30]. The HPCP has been also termed Poisson cluster [30,51], Thomas cluster [20] and homogeneous Thomas cluster process [32]. Finally, to test the joint effects of dispersal limitation and environmental heterogeneity we fitted an IPCP. This process assumes that the spatial pattern is created by dispersal limitation (i.e. as a HPCP), but considers that pattern intensity varies in response to environmental heterogeneity [35]. The IPCP has been also named heterogeneous Thomas process [32,51]. Mathematical descriptions of these three fitted point process models are provided in supplementary material (S1 Text).

To select the process which best fitted the spatial pattern of each species and therefore test the first and second hypotheses we used the Akaike Information Criterion (AIC) [52], based on the sum of residuals and the number of parameters used in the three models [28]. To test if the spatial patterns of species will be faithfully reproduced by any of the considered models, regardless of their dispersal syndrome, we tested for differences in the frequency of IPP, HPCP and IPCP among the five dispersal syndromes by means of an extension of the Fisher’s exact test for larger than 2 ˟ 2 tables (function *fisher*.*test* in R package *stats* [53]. The *P*-value for this test was obtained by 10000 Monte Carlo simulations (*simulate*.*p*.*value* function in the R package *stats*).

For all those species whose patterns were best described by models considering dispersal limitation (i.e. HPCP and IPCP), we assessed the relationships between average cluster size (estimated by σ) and dispersal syndromes by means of a Kruskall-Wallis test. We performed these analyses for the 229 species all together, and for those best fitted by each point pattern process, separately. We also used this test to check whether the parameter σ differed among zoochorous species depending on their fruit size. We performed Wilcoxon rank signed tests to check whether values of σ were smaller for autochorous and anemochorous species all together, than for zoochorous species. All calculations were accomplished in R [53], using *spatstat* [54] and *selectspm* [28] packages.

## Results

**Fig 1:**
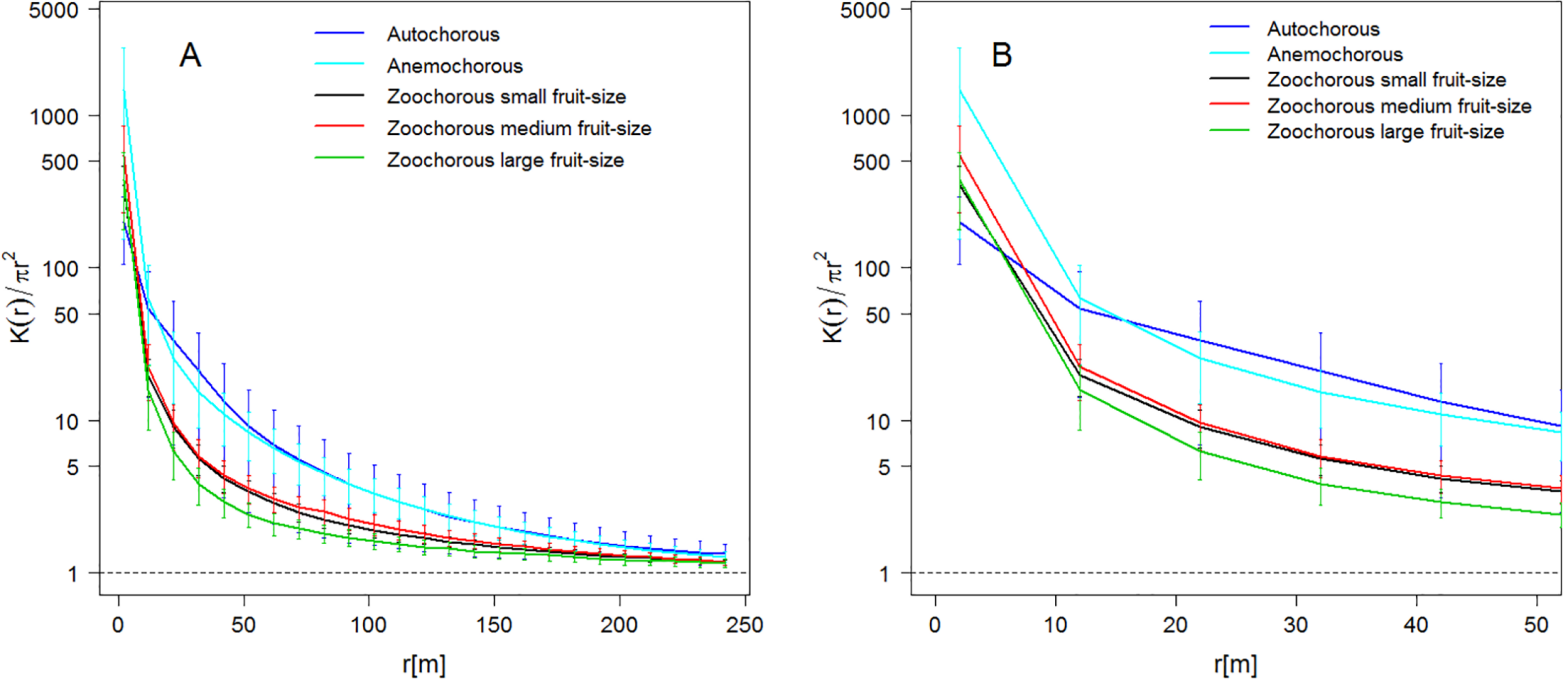
Spatial aggregation by syndrome. The spatial aggregation statistic K(r)/πr^2^ ±1 Standard Error, evaluated each 10 m, up to r = 250 m (A) and up to r = 50 m (B). The dotted line indicates the values of the spatial aggregation statistic for a Poisson random spatial distribution. A species is aggregated at distance r if K(r)/πr^2^ > 1. Zoochorous small fruit-size (< 2 cm), Zoochorous medium fruit-size (2–5 cm), Zoochorous large fruit-size (> 5 cm).

Spatial point patterns of the 229 woody plant species were aggregated (K(*r*)/πr^2^ > 1) at all distances, regardless of their dispersal syndrome (Fig 1A and 1B). Aggregation was very marked for autochorous and anemochorous species at small- and medium-scales (i.e. *r* < 100 m; Fig 1A), but particularly for the anemochorous ones at very fine scales (i.e. *r* < 10 m; Fig 1B).

**Fig 2:**
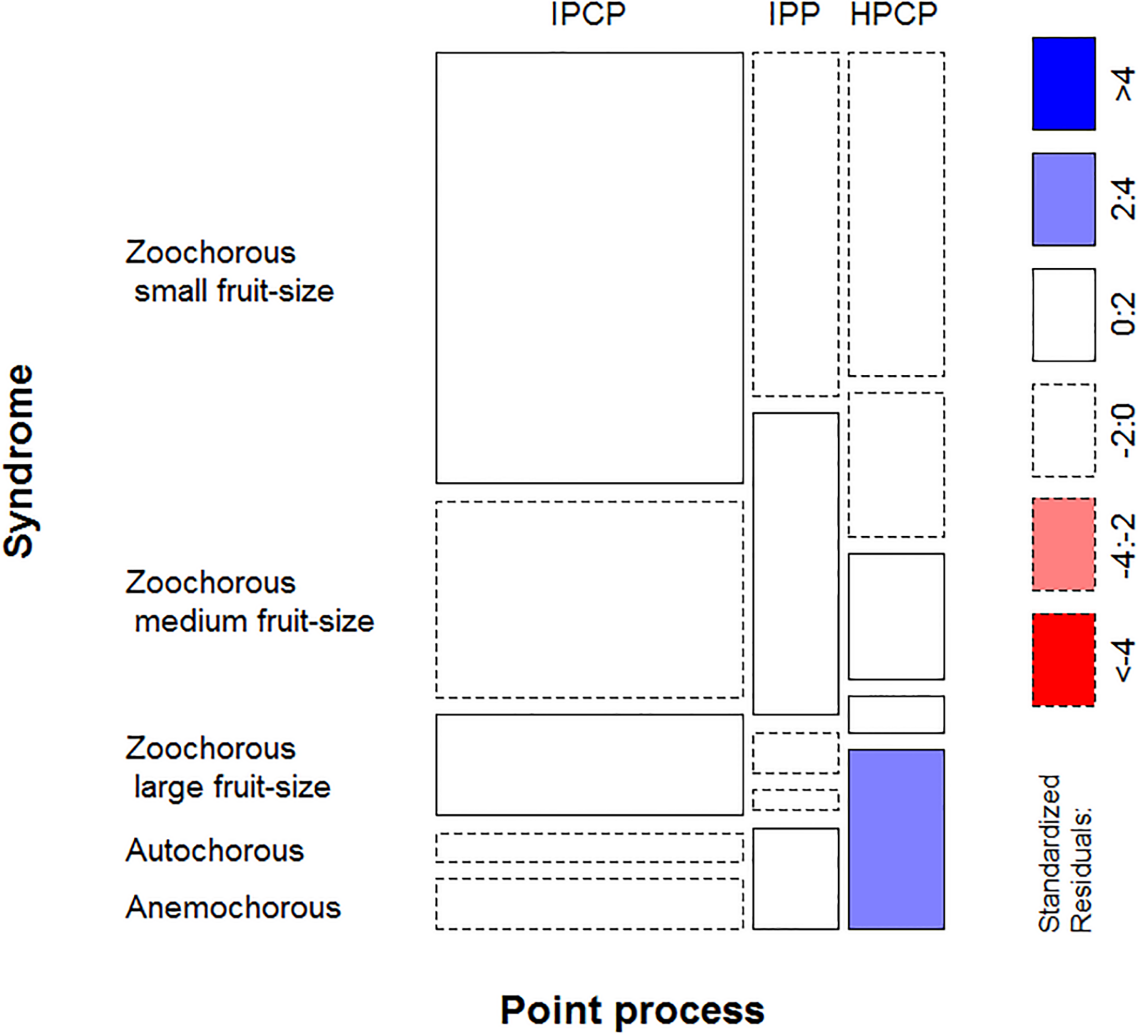
Relationship between point process and dispersal syndrome. Mosaic plot of the frequencies of species showed each of the five dispersal syndromes (autochorous, anemochorous, and zoochorous species with small, medium-size and large fruits) described by each type of point processes; inhomogeneous Poisson processes (IPP), homogeneous Poisson cluster processes (HPCP) and inhomogeneous Poisson cluster processes (IPCP). Frequencies are proportional to the area of the cells. Continuous and discontinuous borders indicate positive and negative deviations of the expected frequencies assuming that dispersal syndromes are independent of the best fitted spatial processes. Cells are colored according to the values of the standardized residuals. If they range 2–4 or are > 2, it indicates a significant relationship between the dispersal syndrome and the best fitting pattern, with *P* < 0.05 and < 0.001, respectively.

Inhomogeneous Poisson cluster process best described the spatial patterns of 63% of the species, whereas HPCP and IPP were the best models for 20% and 17% of them, respectively (Table 1). According to the Fisher’s test results, the frequencies of IPP, HPCP and IPCP as best fitting models significantly differed between the five dispersal types considered (3x5 Fisher’s exact test, *P* = 0.0396). In particular, HPCP significantly prevailed as the best fitting model among anemochorous species (42%) (Fig 2; S2 Table).

**Table 1:** Percentage of species by dispersal syndrome and best fit model.

Point process	IPP		HPCP			IPCP		
Dispersal syndrome	%	*n*	%	*n*	σ (m) ± *SE*	%	*n*	σ (m) ± *SE*
Autochorous	2	1	4	2	11.0 ± 1.34	3	5	8.1 ± 4.5
Anemochorous	12	5	22	10	21.2 ± 4.9	6	9	11.2 ± 2.8
Zoochorous (fruit size < 2 cm)	42	17	40	18	33.6 ± 6.3	53	77	7.7 ± 1.2
Zoochorous (fruit size 2–5 cm)	37	15	18	8	29.3 ± 6.7	24	35	11.0 ± 1.7
Zoochorous (fruit size > 5 cm)	5	2	15	7	51.0 ± 18.1	12	18	9.3 ± 2.7
Total	17	40	20	45	-	63	144	-

Proportion (%) and number (*n*) of species in each dispersal syndrome described by each of the three point pattern processes considered; Inhomogeneous Poisson process (IPP), homogeneous Poisson cluster process (HPCP) and Inhomogeneous Poisson cluster process (IPCP). These processes model the effects of environmental heterogeneity, dispersal limitation and both factors on the spatial point patterns of the species, respectively. Mean dispersal parameter (σ) ± 1 Standard Error (*SE*) for HPCP and IPCP models are also given.

Relationships between average cluster size (estimated by *σ*) and dispersal syndromes were non-significant (Kruskal-Wallis, *χ*^*2*^ = 4.9317, df = 4, *P* = 0.2944). Apparently, autochorous species had the smallest clusters (S1 Fig), but there were not significant differences between their average cluster sizes and those of anemochorous (W = 44, *P* = 0.2087) and zoochorous species (W = 582, *P* = 0.9088). Besides, there were no significant differences between the average cluster sizes of zoochorous species, and between those of autochorous and anemochorous species (W = 1733.5, *P* = 0.1368).

## Discussion

Our findings clearly corroborate that aggregation is the norm for conspecific spatial patterns in this forest [11,30] since the spatial patterns of 229 woody plant species present in the Barro Colorado Island Forest Dynamics Plot (BCI) in 1995 were aggregated, regardless of their dispersal syndrome. In most species this aggregation occurred over a long range of distances, which suggests that it is not only due to the effect of plant-plant interactions or dispersal limitation but to environmental heterogeneity [55]. The spatial patterns of around 80% of the species were described by inhomogeneous processes (i.e. IPCP and IPP; Table 1), which indicates that environmental heterogeneity affects the spatial signatures of species independently of the dispersal syndrome and at ample range of spatial scales. These facts point towards a high relative importance of environmental heterogeneity in determining the spatial structure of individual populations in BCI.

Our first hypothesis was confirmed; the spatial patterns of 63% of the species were best described by an IPCP, which suggests that they are not entirely determined by environmental heterogeneity, but by their joint effects with dispersal limitation. These results concur with others found in other temperate [34], subtropical [32] and tropical dry forests [28]. In some of these studies the relative role of environmental heterogeneity versus dispersal limitation on conspecific spatial point patterns have been explained by the large topographic heterogeneity within the plots [32,34]. This explanation, however, does not seem to apply in BCI since the majority of species in this forest are not significantly associated with topographically defined habitat types [9,29,56]. On the contrary, in BCI tropical forest, where severe droughts associated with El Niño events periodically occur [57], species distributions are largely determined by their drought-sensitivity with respect to local water availability [58]. They are also strongly associated with changes in underlying soil chemical properties [20,59], reinforcing the idea that environmental heterogeneity plays a crucial role to determine the spatial patterns of tree species in BCI, that probably operates through species-specific responses. Our approach, which estimated the environmental heterogeneity surfaces not as a function of some environmental covariates [33] but from the pattern of trees themselves, shows that the spatial scale at which they occur varies from species to species (S1 Table).

As predicted in our second hypothesis, HPCP significantly prevailed as the best fitting model for anemochorous species, which showed the strongest aggregation at smallest scales (i.e. *r* < 10 m). These results indicate that spatial patterns of anemochorous species are strongly influenced by dispersal limitation. Several studies have demonstrated that seed dispersal distance of anemochorous species depends on propagule morphology (which affects their aero-dynamic properties) and forest structure (which affects wind circulation) [8,41,60]. Seed dispersal of anemochorous species in BCI is critically affected by small-scale air turbulence and a large amount of them arrive at tree-fall gaps [61]. Indeed, most anemochorous species in this forest are pioneer trees which largely depend on gaps to regenerate [61,62]. It is also important to note that most tree fall gaps are small (< 50 m^2^) and are homogeneously distributed throughout the plot [62,63], therefore, as germination and establishment of pioneer species (which are mainly anemochorous) necessarily occurs in gaps, their distribution will mimic that of the latter. Light availability, soil moisture and nutrient contents are higher in gaps than in the surrounding areas all over the forest [64], which points toward an overlap between the scales at which dispersal limitation and environmental heterogeneity (both restricted to gaps) occur. All these observations, together with our findings, suggest that dispersal limitation has a major influence on the spatial patterns of Neotropical pioneer trees [14]. Note also that HPCP was the second best fitting model for zoochorous species with small fruit size (< 2 cm). These tree species, many of them typical of the understory of tropical forests, are mostly dispersed by small birds and dropped almost exclusively in tree-fall gaps [65].

Our results suggest that HPCP models adequately describe the spatial patterns not only of anemochorous species, but of all species whose seed dispersal is strongly restricted to tree-fall gaps. Nevertheless, it does not mean that the spatial distribution of these species does not respond to environmental heterogeneity, simply that in this case, the effects of heterogeneity represented by gaps (which are very small sized perturbations) appear at very fine spatial scale not included within the set of bandwidths employed to describe the environmental heterogeneity in our study. Note also that 20% of the anemochorous species (e.g. *Ceiba pentandra*, *Lonchocarpus heptaphyllus*, *Tabebuya rosea*), were best fitted by an IPP, which indicates that their spatial patterns are determined by environmental heterogeneity. This result is surprising since a Poisson process (homogeneous or inhomogeneous) is equivalent to a Poisson cluster process (e.g. HPCP and IPCP) in which the average cluster size (estimated by σ) is larger than the scale of the plot. This result could therefore imply the existence of secondary dispersal or strong environmental filtering for such species at the scale of the BCI plot [28]. The evidence that some species are better adjusted by HPCP does not imply absence of environmental heterogeneity in the formation of the spatial pattern. Seed dispersal sets the template for tree distribution [51], and this template is subject to the effects from variety of environmental filtering.

The absence of significant relationships between dispersal syndrome and average cluster size for the subset of 189 species with homogeneous and inhomogeneous Poisson cluster as their best fitted models, also confirms our third hypothesis. These results contrast with those of [30], which pointed toward strong and significant relationships between dispersal syndrome and average cluster size. These contrasting results could be explained because these authors fitted the spatial patterns of all species to a HPCP, taking into account just the effects of dispersal limitation. Our findings demonstrate that environmental heterogeneity blurs the spatial patterns generated by seed dispersal in the BCI plot at a wide range of spatial scales and independently of the dispersal syndrome. This non-significant relationship could be explained by factors as landscape elements which trap seeds in the forest [18], and plant—plant interactions that modify the spatial pattern generated by the joint effects of dispersal limitation and environmental heterogeneity [66]. Both factors seem to operate in our case, since tree-fall gaps, where a large amount of wind-dispersed seeds end up, are particularly common in BCI in comparison with other tropical forests [67]. Besides, density-dependent mortality at the early stages of tree development is prevalent in this plot [68,69]. Moreover, density-dependent effects among saplings are more prevalent at smaller spatial scales (*r* < 5 m) [70] than those at which seed dispersal and environmental heterogeneity occur (*r* = 10–50 m). All this evidence suggests that dispersal limitation, environmental heterogeneity and also plant-plant interactions act sequentially, increasing regularity in conspecific spatial patterns of this forest. This sequence has strong implications for species coexistence, since conspecific clustered patterns enhance the impact of intra- vs. inter-specific interactions, allowing competitively inferior species to perform better and avoid exclusion [17,71]. Promoting trade-offs between colonization and competition abilities among the species mediated by a relatively intense tree fall and gap dynamics [72].

Interestingly, whilst autochorous species had the smallest mean cluster size (S1 Fig), the point process model best describing the spatial patterns of all species (i.e. IPCP) did not corroborate that the smallest cluster sizes should belong to the autochorous species (Table 1). Consequently, the analysis did not provide support for our fourth hypothesis. Worth to note that the number of autochorous species is very low, which could hinder the detection of this expectation. Our fifth hypothesis was also not confirmed since average cluster sizes of zoochorous species were not significantly larger than those of autochorous and anemochorous species. Both results could be attributed to the fact that zoochorous species with small fruit size, which are shade-tolerant or have intermediate light requirements, are more abundant in small gaps, whereas anemochorous species, which are mainly pioneer and light demanding, are more abundant in large gaps [73]. The higher abundance of anemochorous species in large gaps might explain also the fact that its average cluster size was larger than that of zoochorous species with small (< 2 m) and medium (2–5 cm) fruit size.

Concluding, our results support the hypothesis that local conspecific spatial patterns in tropical forests such as that on Barro Colorado Island (Panama) depend on dispersal mode [30], but not exclusively so. Rather, they are explained by the joint effects of dispersal limitation and environmental heterogeneity as suggested by [22] and [33]. The relative importance of these two processes in determining conspecific spatial patterns is rather similar, although dispersal limitation is a major factor for anemochorous species. Moreover, the filter exerted by the abiotic environment occurs at similar spatial scales as dispersal limitation, and is as marked in BCI as in other forests with larger topographic gradients [28,32,33,36]. This definitively challenges the long standing idea that dispersal limitation is critical at fine scales [74] whereas environmental heterogeneity at medium and large ones [75–77].

Our work emphasizes the benefits of fitting several point process models that embody the relevant underlying biological mechanisms when assessing conspecific spatial patterns in fully-mapped plots [28,33]. It also highlights the strong potential of spatially explicit techniques to generate testable hypotheses on the main driving assembly processes and species coexistence in plant communities [3,35,78]. Although other studies have identified the importance of habitat heterogeneity at fine scales without being able to associate it with other processes, our results help fill the void about the role of habitat heterogeneity in the spatial distribution of species. In further studies, it would be desirable to check whether the point processes best describing the conspecific spatial patterns and the absence of significant relationships between dispersal syndrome and average cluster size remain over the 26 years of available censuses in this and others mapped plots. It would be also interesting to check if these variations are related to changes in overall climatic conditions (e.g. El Niño events in 1983 and 1998) [70].

## Supporting information

S1 Fig Mean cluster size by dispersal syndrome. Mean cluster size (σ) ± Standard Error of the five types of species according to their dispersal syndromes (autochorous, anemochorous, and zoochorous species with small, medium-size and large fruits). Black dots and lines indicate resulting values for 189 species by selecting the best fitting model among homogeneous or inhomogeneous Poisson cluster processes (HPCP and IPCP).
(PNG)

S1 Table Bandwidth for inhomogeneous processes. Number (*n*) of species in each dispersal syndrome described by each of the two inhomogeneous point processes considered; Inhomogeneous Poisson process (IPP) and Inhomogeneous Poisson cluster process (IPCP). These processes model the effects of environmental heterogeneity, and the joint effect between dispersal limitation and environmental heterogeneity on the spatial point patterns of the species, respectively. Mean bandwidth parameter ± 1 Standard Error (SE) for IPP and IPCP models are also given.
(PDF)

S2 Table Dispersal syndrome and spatial point process fitted to each species. Best fit: spatial point process fitted for each species. Inhomogeneous Poisson process (IPP), homogeneous Poisson cluster process (HPCP); inhomogeneous Poisson cluster process (IPCP). Dispersal: dispersal syndrome assigned to each species. Animal1: zoochorous (fruit size < 2 cm); aninal2: zoochorous (fruit size 2–5 cm); animal3: zoochorous (fruit size > 5 cm).
(PDF)

S1 Text Spatial point processes. Mathematical details of the three spatial point processes: Inhomogeneous Poisson Processes (IPP), Homogeneous Poisson cluster process (HPCP), Inhomogeneous Poisson cluster process (IPCP).
(DOCX)
