# Supplementary figures and images for: Environmental heterogeneity blurs the signature of dispersal syndromes on spatial patterns of woody species in a moist tropical forest
Source: PLoS One. 2018 Feb 16;13(2):e0192341. doi: 10.1371/journal.pone.0192341 (PMC5815593; doi:10.1371/journal.pone.0192341)

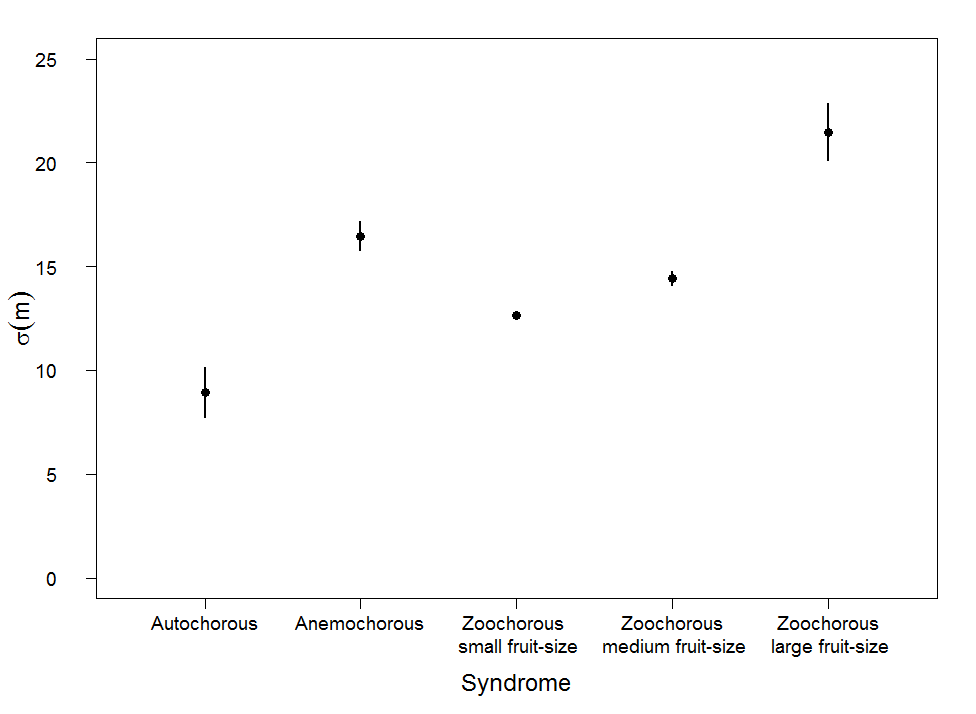

Supplement: S1 Fig — Mean cluster size (σ) ± Standard Error of the five types of species according to their dispersal syndromes (autochorous, anemochorous, and zoochorous species with small, medium-size and large fruits). Black dots and lines indicate resulting values for 189 species by selecting the best fitting model among homogeneous or inhomogeneous Poisson cluster processes (HPCP and IPCP). (PNG) [file pone.0192341.s001.png]
