# Supplementary material for: Environmental heterogeneity blurs the signature of dispersal syndromes on spatial patterns of woody species in a moist tropical forest
Source: PLoS One. 2018 Feb 16;13(2):e0192341. doi: 10.1371/journal.pone.0192341 (PMC5815593; doi:10.1371/journal.pone.0192341)
Supplement: S1 Table — Number (n) of species in each dispersal syndrome described by each of the two inhomogeneous point processes considered; Inhomogeneous Poisson process (IPP) and Inhomogeneous Poisson cluster process (IPCP). These processes model the effects of environmental heterogeneity, and the joint effect between dispersal limitation and environmental heterogeneity on the spatial point patterns of the species, respectively. Mean bandwidth parameter ± 1 Standard Error (SE) for IPP and IPCP models are also given. (PDF) [file pone.0192341.s002.pdf]

S1 Table. Bandwidth for inhomogeneous processes. Number (*n*) of species in each dispersal syndrome described by each of the two inhomogeneous point processes considered; Inhomogeneous Poisson process (IPP) and Inhomogeneous Poisson cluster process (IPCP). These processes model the effects of environmental heterogeneity, and the joint effect between dispersal limitation and environmental heterogeneity on the spatial point patterns of the species, respectively. Mean bandwidth parameter ± 1 Standard Error (SE) for IPP and IPCP models are also given.

	**IPP**		**IPCP**	
**Dispersal syndrome**	***n***	**Bandwidth (m)**	***n***	**Bandwidth (m)**
Autochorous	17	51.0	77	223.8 ± 15.1
Anemochorous	15	70.2 ± 17.5	35	218.6 ± 13.4
Zoochorous (fruit size < 2 cm)	2	59.9 ± 10.3	18	198.4 ± 4.5
Zoochorous (fruit size 2-5 cm)	1	79.3 ± 17.8	5	207.0 ± 7.4
Zoochorous (fruit size > 5 cm)	5	71.0 ± 40.0	9	181.1 ± 8.0
