# Supplementary material for: Environmental heterogeneity blurs the signature of dispersal syndromes on spatial patterns of woody species in a moist tropical forest
Source: PLoS One. 2018 Feb 16;13(2):e0192341. doi: 10.1371/journal.pone.0192341 (PMC5815593; doi:10.1371/journal.pone.0192341)
Supplement: S2 Table — Best fit: spatial point process fitted for each species. Inhomogeneous Poisson process (IPP), homogeneous Poisson cluster process (HPCP); inhomogeneous Poisson cluster process (IPCP). Dispersal: dispersal syndrome assigned to each species. Animal1: zoochorous (fruit size < 2 cm); aninal2: zoochorous (fruit size 2–5 cm); animal3: zoochorous (fruit size > 5 cm). (PDF) [file pone.0192341.s003.pdf]

Species	Best fit	Dispersal
Acalypha diversifolia	IPCP	autochorous
Acalypha macrostachya	HPCP	autochorous
Adelia triloba	HPCP	autochorous
Aegiphila panamensis	HPCP	animal1
Alchornea costaricensis	HPCP	animal1
Alibertia edulis	IPCP	animal2
Allophylus psilospermus	IPCP	animal1
Alseis blackiana	HPCP	anemochorous
Amaioua corymbosa	HPCP	animal1
Anacardium excelsum	HPCP	animal3
Anaxagorea panamensis	HPCP	autochorous
Andira inermis	HPCP	animal3
Annona acuminata	HPCP	animal2
Annona spraguei	IPCP	animal2
Apeiba membranacea	HPCP	animal2
Apeiba tibourbou	IPP	animal2
Aspidosperma spruceanum	HPCP	anemochorous
Astrocaryum standleyanum	IPCP	animal2
Astronium graveolens	HPCP	anemochorous
Attalea butyracea	IPP	animal2
Bactris barronis	IPCP	animal1
Bactris coloniata	HPCP	animal2
Bactris major	HPCP	animal2
Beilschmiedia pendula	HPCP	animal3
Brosimum alicastrum	IPP	animal1
Calophyllum longifolium	IPP	animal2
Capparis frondosa	IPCP	animal3
Casearia aculeata	IPCP	animal1
Casearia arborea	HPCP	animal1
Casearia commersoniana	IPCP	animal1
Casearia guianensis	HPCP	animal1
Casearia sylvestris	HPCP	animal1
Cassipourea elliptica	IPP	animal1
Cavanillesia platanifolia	IPP	anemochorous
Cecropia insignis	HPCP	animal3
Cecropia obtusifolia	IPCP	animal3
Ceiba pentandra	IPP	anemochorous
Celtis schippii	IPP	animal1
Cespedesia spathulata	HPCP	anemochorous
Cestrum megalophyllum	IPCP	animal1
Chamguava schippii	HPCP	animal1
Chrysochlamys eclipes	HPCP	animal1
Chrysophyllum argenteum	IPP	animal2
Chrysophyllum cainito	IPP	animal2
Cinnamomum triplinerve	IPCP	animal1
Clidemia dentata	HPCP	animal1
Coccoloba coronata	IPCP	animal1

Coccoloba manzinellensis	IPCP	animal1
Conostegia cinnamomea	HPCP	animal1
Cordia alliodora	HPCP	anemochorous
Cordia bicolor	IPP	animal1
Cordia lasiocalyx	IPCP	animal1
Coussarea curvigemmia	HPCP	animal1
Croton billbergianus	HPCP	autochorous
Cupania latifolia	HPCP	animal1
Cupania rufescens	HPCP	animal1
Cupania seemannii	IPP	animal1
Dendropanax arboreus	HPCP	animal1
Desmopsis panamensis	HPCP	animal1
Diospyros artanthifolia	HPCP	animal2
Dipteryx oleifera	IPP	animal2
Drypetes standleyi	IPCP	animal1
Elaeis oleifera	HPCP	animal2
Erythrina costaricensis	HPCP	animal1
Erythroxylum macrophyllum	IPP	animal1
Erythroxylum panamense	HPCP	animal1
Eugenia coloradoensis	HPCP	animal2
Eugenia galalonensis	IPP	animal1
Eugenia nesiotica	IPP	animal2
Eugenia oerstediana	IPP	animal1
Faramea occidentalis	IPCP	animal1
Ficus costaricana	HPCP	animal1
Ficus obtusifolia	HPCP	animal2
Ficus tonduzii	HPCP	animal2
Garcinia intermedia	IPP	animal2
Garcinia madruno	HPCP	animal2
Genipa americana	IPP	animal3
Guapira standleyana	IPP	animal1
Guarea bullata	IPP	animal1
Guarea grandifolia	HPCP	animal2
Guarea guidonia	IPP	animal2
Guatteria dumetorum	IPP	animal1
Guazuma ulmifolia	HPCP	animal2
Guettarda foliacea	HPCP	animal2
Gustavia superba	HPCP	animal3
Hamelia axillaris	HPCP	animal1
Hampea appendiculata	HPCP	animal2
Hasseltia floribunda	HPCP	animal1
Heisteria acuminata	HPCP	animal1
Heisteria concinna	HPCP	animal1
Herrania purpurea	IPP	animal3
Hieronyma alchorneoides	HPCP	animal1
Hirtella americana	HPCP	animal2
Hirtella triandra	IPCP	animal2
Hura crepitans	IPP	autochorous

Hybanthus prunifolius	HPCP	autochorous
Inga acuminata	IPCP	animal3
Inga cocleensis	HPCP	animal3
Inga goldmanii	HPCP	animal3
Inga laurina	IPP	animal3
Inga marginata	IPP	animal3
Inga nobilis	IPP	animal3
Inga pezizifera	HPCP	animal3
Inga punctata	HPCP	animal3
Inga ruiziana	HPCP	animal3
Inga sapindoides	IPCP	animal3
Inga spectabilis	HPCP	animal3
Inga thibaudiana	IPP	animal3
Inga umbellifera	IPCP	animal3
Jacaranda copaia	HPCP	anemochorous
Lacistema aggregatum	IPP	animal1
Lacmellea panamensis	HPCP	animal2
Laetia procera	IPCP	animal1
Laetia thamnia	HPCP	animal2
Licania hypoleuca	HPCP	animal2
Licania platypus	HPCP	animal3
Lindackeria laurina	IPCP	animal1
Lonchocarpus heptaphyllus	IPP	anemochorous
Luehea seemannii	IPCP	anemochorous
Macrocnemum roseum	HPCP	anemochorous
Malpighia romeroana	HPCP	animal1
Maquira guianensis	IPP	animal1
Marila laxiflora	HPCP	anemochorous
Maytenus schippii	HPCP	animal1
Miconia affinis	IPCP	animal1
Miconia argentea	IPCP	animal1
Miconia elata	IPCP	animal1
Miconia hondurensis	IPCP	animal1
Miconia nervosa	HPCP	animal1
Mosannona garwoodii	HPCP	animal1
Mouriri myrtilloides	HPCP	animal1
Myrcia gatunensis	HPCP	animal1
Myrospermum frutescens	HPCP	anemochorous
Nectandra cissiflora	HPCP	animal1
Nectandra lineata	IPCP	animal1
Nectandra purpurea	HPCP	animal1
Neea amplifolia	IPCP	animal1
Ocotea cernua	IPCP	animal1
Ocotea oblonga	IPP	animal1
Ocotea puberula	IPP	animal1
Ocotea whitei	HPCP	animal2
Oenocarpus mapora	IPCP	animal2
Ormosia coccinea	HPCP	animal2

Ormosia macrocalyx	IPP	animal2
Ouratea lucens	HPCP	animal1
Pachira sessilis	HPCP	anemochorous
Palicourea guianensis	IPCP	animal1
Pentagonia macrophylla	HPCP	animal2
Perebea xanthochyma	HPCP	animal1
Picramnia latifolia	IPCP	animal1
Piper aequale	HPCP	animal1
Piper arboreum	HPCP	animal3
Piper colonense	HPCP	animal1
Piper cordulatum	IPCP	animal2
Piper perlasense	HPCP	animal1
Piper reticulatum	HPCP	animal1
Platymiscium pinnatum	HPCP	anemochorous
Platypodium elegans	IPP	anemochorous
Posoqueria latifolia	IPP	animal2
Poulsenia armata	HPCP	animal2
Pourouma bicolor	HPCP	animal1
Pouteria reticulata	IPP	animal2
Pouteria stipitata	IPP	animal2
Prioria copaifera	HPCP	animal3
Protium costaricense	IPP	animal1
Protium panamense	HPCP	animal2
Protium tenuifolium	IPP	animal1
Psidium friedrichsthalianum	IPCP	animal2
Psychotria acuminata	IPCP	animal1
Psychotria chagrensis	HPCP	animal1
Psychotria deflexa	HPCP	animal1
Psychotria graciliflora	HPCP	animal1
Psychotria grandis	IPCP	animal1
Psychotria horizontalis	IPCP	animal1
Psychotria limonensis	HPCP	animal1
Psychotria marginata	IPCP	animal1
Pterocarpus rohrii	IPP	anemochorous
Quararibea asterolepis	IPP	animal1
Quassia amara	IPCP	animal1
Randia armata	IPP	animal2
Rinorea sylvatica	HPCP	autochorous
Sapium glandulosum	HPCP	animal1
Senna dariensis	HPCP	animal1
Simarouba amara	IPP	animal1
Siparuna guianensis	HPCP	animal1
Siparuna pauciflora	HPCP	animal1
Sloanea terniflora	HPCP	animal1
Socratea exorrhiza	HPCP	animal2
Solanum hayesii	HPCP	animal1
Sorocea affinis	IPCP	animal1
Spachea membranacea	HPCP	animal1

Spondias mombin	HPCP	animal2
Spondias radlkoferi	HPCP	animal2
Sterculia apetala	IPP	animal1
Swartzia simplex	HPCP	animal2
Symphonia globulifera	HPCP	animal2
Tabebuia guayacan	HPCP	anemochorous
Tabebuia rosea	HPCP	anemochorous
Tabernaemontana arborea	IPCP	animal2
Tachigali versicolor	HPCP	anemochorous
Talisia nervosa	HPCP	animal2
Talisia princeps	HPCP	animal2
Terminalia amazonia	HPCP	anemochorous
Terminalia oblonga	HPCP	anemochorous
Tetragastris panamensis	IPP	animal2
Tetrathylacium johansenii	HPCP	animal2
Theobroma cacao	HPCP	animal3
Thevetia ahouai	HPCP	animal2
Trattinnickia aspera	HPCP	animal1
Trema micrantha	HPCP	animal1
Trichilia pallida	IPCP	animal1
Trichilia tuberculata	HPCP	animal1
Triplaris cumingiana	IPCP	anemochorous
Trophis caucana	HPCP	animal1
Trophis racemosa	HPCP	animal1
Turpinia occidentalis	IPP	animal1
Unonopsis pittieri	HPCP	animal1
Urera baccifera	HPCP	animal1
Virola multiflora	HPCP	animal2
Virola sebifera	IPP	animal2
Virola surinamensis	IPP	animal2
Vismia baccifera	IPCP	animal1
Vochysia ferruginea	HPCP	anemochorous
Xylopia macrantha	HPCP	animal1
Xylosma oligandra	IPCP	animal1
Zanthoxylum acuminatum	IPP	animal1
Zanthoxylum ekmanii	IPCP	animal1
Zanthoxylum panamense	IPP	animal1
Zuelania guidonia	HPCP	animal3
