# Supplementary material for: Environmental heterogeneity blurs the signature of dispersal syndromes on spatial patterns of woody species in a moist tropical forest
Source: PLoS One. 2018 Feb 16;13(2):e0192341. doi: 10.1371/journal.pone.0192341 (PMC5815593; doi:10.1371/journal.pone.0192341)
Supplement: S1 Text — Mathematical details of the three spatial point processes: Inhomogeneous Poisson Processes (IPP), Homogeneous Poisson cluster process (HPCP), Inhomogeneous Poisson cluster process (IPCP). (DOCX) [file pone.0192341.s004.docx]

**S1 Text. Mathematical details of the three spatial point processes**

**Fitting models of spatial point processes**

Spatial point pattern description is based on the Ripley’s *K*-function [1,2]. For a homogeneous pattern with intensity *λ*, *λK(r)* is the expected number of points within circle of radius *r* around any point of the pattern. In case of inhomogeneous patterns, heterogeneous *K*-function (*K_I_(r)*) is used [3].

Model fitting is based on minimizing a discrepancy measure between the observed and the theoretical *K*-function of the assumed point process, method known as “minimum contrast” or “least squares” estimation.

As a discrepancy measure we employed the one proposed by [4]:

$$D\left( \theta\right)= \int_{0}^{r_{0}} w\left( r \right)\left[ \left\{ \hat{K}\left( r \right) \right\}^{c}-\left\{ K\left( r;\theta\right) \right\}^{c} \right]^{2}dr,$$

where *r* is the vector of *r* values at which the *K*- function is estimated, $\hat{K}(r)$ and *K(r;θ)* are respectively the empirical and the theoretical (with parameters *θ*) *K*-functions, *r_0_* is the maximum radius *r* for which the *K-* functions have been computed, *w(r)* is the weighting function and the constant *c* are employed to control the sampling fluctuations in $\hat{K}(r)$. We followed the recommendations of [5] and set *w(r)* = 1 and *c* = 0.25. We also set *r_0_* = 250 m, i.e, ¼ of the larger side of the plot.

**Inhomogeneous Poisson Process (IPP)**

Is a non-stationary point process that share with homogeneous Poisson process the independence of points but their intensity is not constant but instead varies from place to place according to an intensity function *λ*(*u*) which, in ecological studies, it is assumed to depend on environmental heterogeneity (e.g., [6-8]). To characterize IPP, we employed the inhomogeneous *K*-function [3]. It is estimated as

$$\hat{K}_{I}\left( r \right)=\frac{1}{A}\sum_{i=1}^{n} \sum_{j\neq i} \frac{w_{ij}}{{\lambda(x}_{i}){\lambda(x}_{j})}I(d_{ij}<r)$$

where the expected value of the inhomogeneous *K*-function for an IPP is *K_I_*$\left( r \right)=\pi r^{2}$.

The IPP assumes the pattern formation in two stages, first a homogenous Poisson pattern is generated, and then the points are retained with a probability proportional to the surface λ(x), which describes the environmental heterogeneity. In the absence of values of environmental variables, that surface is estimated with a kernel function. This process can be used to examine the interaction effects between tree density and environmental factors [9].

Here intensity function was estimated by means of kernel smoothing [7,10], which compute the intensity at location *u* as $\hat{\lambda}(u)=e(u)\sum_{i=1}^{n} \kappa(u-x_{i})$, where κ(*u*) is an arbitrary kernel function and *e*(*u*) is an edge correction term. We employed the two-dimensional Gaussian kernel as smoothing function, i.e.,

$$\kappa\left( u-x_{i} \right)=\frac{1}{2\pi\sigma^{2}} e^{-\frac{1}{2}\left( \frac{u-x_{i}}{\sigma} \right)^{2}}$$

Note that here σ represents the standard deviation of the smoothing Gaussian kernel, and we will refer to it hereafter as *σ_λ_*. We employed the function density.ppp() in spatstat [11], with the argument at=”points”, to estimate the intensity at each point of the pattern [i.e, to estimate the individual *λ(x_i_)* terms in the formula for the estimation of the inhomogeneous K]. Choosing the bandwidth of the kernel for estimation of an intensity surface is based on biological criteria, i.e, selecting a bandwidth larger than the scale at which second order effects show up [6,7].

In some other studies, environmental covariates have been employed to fit intensity surfaces (e.g. [12, 13]). However, in our opinion such procedure is limited by the availability of maps of the relevant environmental covariates ([8] pp. 129, and [14]), by its relationship with the processes generating the intensity and by the resolution of those maps. Although there exists maps of environmental covariates for BCI, either they are unable to completely explain the inhomogeneity for all BCI species or, in the best of cases, they provide worst estimates of intensity surfaces than kernel smoothing of the point data themselves. We explicitly make the assumption that kernel smoothed surfaces “allow the detection of potential gradients in the intensity that point to underlying (environmental) heterogeneity” (as [6, 7] and [8] pp. 94), i.e., that inhomogeneity is the consequence of environmental heterogeneity an therefore, if the inhomogeneous model fits the spatial pattern of some species better than an homogeneous one we could figure the relative importance of environmental heterogeneity.

We estimated for each species a set of 56 different intensity surfaces, using Gausian kernels varying from *σ_λ_* =15 to *σ_λ_* =125 m in 1 m steps (these are equivalents to "bandwidths" from 30 to 250 m; [15]). In our approach, model fitting of the IPP model involves choosing the value for the standard deviation *σ_λ_* of the smoothing Gaussian kernel that minimize the discrepancy measure *D(θ)*. For each plant species, we computed the discrepancy measure *D(θ)* between the theoretical K_I_ function for an IPP (i.e., *K_I_(r) = πr^2^*) and a set of different empirical K_I_ functions (computed from each of the 56 different estimated intensity surfaces). We selected the *σ_λ_* value and the corresponding K_I_ function which produced the smallest *D(θ)*.

**Homogeneous Poisson cluster process (HPCP)**

Poisson cluster processes generate non-independent (clustered) points in a two-step process. First, a Poisson process of "parent" points is generated with intensity *ρ*. Then, each parent point produces "offspring"; the number of offspring per parent follows a Poisson distribution, and their locations are independent and isotropically normally distributed around the parent tree, with mean zero and standard deviation *σ*. The theoretical K function for a HPCP is

$K\left( r; \rho,\sigma\right)=\pi r^{2}+\frac{1-e^{\left( -r^{2}/4\sigma^{2} \right)}}{\rho}$ ,

The empirical K function is computed as for Poisson process. Model fitting involves iteratively choosing the parameters *ρ* and *σ* that minimize the discrepancy measure *D(θ)*. We minimized *D(θ)* with the optim() function in R using the algorithm of [16] (method = "L-BFGS-B" in optim), which allows box constraints on the parameter space. This avoids getting unrealistic values for parameters, a not unusual result when using unconstrained optimization to fit Poisson cluster models. We constrained the parameter space between *1/A* and *n/A* for *ρ* (i.e., between only one cluster and *n* clusters) and between 1/10 m and 4*r_0_* m for *σ* [i.e., between one tenth of the *r* interval and 4 times the maximum radius employed to compute K(r), which equals the maximum size of each side of the plot.

The parameters *ρ* and *σ* are usually adjusted by comparing the empirical K-funtion with the theoretical K-function using the least contrast method [5,17]. Thus, the aggregation of the pattern is quantified by ρ (average number of groups), and the estimated mean size of the groups is given by *σ*.

The HPCP considers the distribution of offspring as a limited dispersion function and assumes homogeneity; however the characteristics of this condition may not be accomplish by many species due to environmental heterogeneity and habitat association [6,18,19].

**Inhomogeneous Poisson cluster process (IPCP)**

The IPCP is suitable to evaluate the joint effect of limited dispersion and habitat heterogeneity [13,20]. Unlike HPCP, the number of offspring per parent is not a constant, but should be estimated through a function of spatially heterogeneous intensity [21,22].

As an alternative of IPP, is an extension of the HPCP, where it is assumed that the distribution of points, in addition to clustered, is inhomogeneous. The expectation for the inhomogeneous K-function of an IPCP is

$K_{I}\left( r \right)=\pi r^{2}+\frac{1-e^{\left( -r^{2}/4\sigma^{2} \right)}}{\rho}$,

and the empirical $\hat{K}_{I}(r)$ function is computed as for an inhomogeneous point pattern. Fitting an IPCP model implies getting the values of *ρ*, *σ* and of the standard deviation of the Gaussian smoothing kernel (*σ_λ_*) which minimize *D(θ)*. Instead of assign the three parameters directly in a function to be optimized numerically; we fitted the model for each species in a two-step process: first, we computed 56 inhomogeneous K_I_ functions based on the 56 different intensity surfaces (the same inhomogeneous K functions estimated before for the IPP fitting). We then selected the values of *ρ* and *σ* that minimized the discrepancy measure *D(θ)* between each of the 56 K_I_ functions and their theoretical expectation, using the R function optim() in the same way as for PCP’s. We obtained 56 *D(θ)* values for each species. Finally we selected the σ, *ρ* and the standard deviation of the smoothing kernel (*σ_λ_*) which produced the smallest *D(θ)*.

Both homogeneous and inhomogeneous K-functions were estimated using Ripley’s isotropic edge correction [1]. All the functions were estimated up to 250 m with steps of 1 m. Models were fitted with code based on “selectspm” R package [23].

**Selection of best fit model**

To get a balance between optimal fitting and model complexity, we selected the best model for each species based on Akaike Information Criterion (AIC). AIC is defined as

$$AIC= -2\ln\left( L\left( \hat{\theta} \right) \right)+ 2p$$

were $L\left( \hat{\theta} \right)$ is the maximized likelihood of the model and $\hat{\theta}$ the vector of *p* fitted parameters [24]. Following [25,26] this can be estimated as

$$\hat{AIC}= \left[ n \ln\left( \frac{2\pi}{n} \right)+n+2 \right] n\ln R+2p$$

where n is the number of observations [in our case, the number of r intervals where K(r) has been estimated] and R the mean of the square residuals between the observed and the fitted values [in our case, *R = D(θ) /n*].

We clarify that R is not a sum of squares residuals like some publications [13, 25]. Since the term in square brackets is constant, we should compute only $\hat{A}= n\ln R+2p$ [25,26]. Following the recommendations of [20], to avoid bias caused by small sample size. For each species we estimated the small AIC value as:

$$\hat{AIC}_{c}= \hat{A}+ \frac{2p(p+1)}{n-p-1}$$

Although kernel smoothing estimation is a non-parametric method of density estimation, we are not fitting density (i.e., intensity) models but K-functions (i.e., theroretical K-functions to empirical K functions). Therefore, in this context, the consideration of an intensity surface represents just one extra-element of information and adds one parameter in the computation of AIC, i.e., the procedure remains parametric.

**Additional References**

1. Ripley BD. 1977 Modelling spatial patterns. *Journal of the Royal Statistical Society. Series B (Methodological)* **39**, 172–212.
2. Illian J, Penttinen A, Stoyan H, Stoyan D. 2008 *Statistical analysis and modelling of spatial point patterns*. Chichester UK: Wiley.
3. Baddeley AJ, M∅ller J, Waagepetersen R. 2000 Non- and semiparametric estimation of interaction in inhomogeneous point patterns. *Statistica Neerlandica* **54**, 329–350.
4. Diggle PJ. 2013 *Statistical Methods for Spatial and Spatio-Temporal Point Patterns*. 3rd ed. Boca Raton: CRC Press.
5. Diggle PJ. 2003 *Statistical analysis of spatial point patterns*. 2nd ed. London: Arnold.
6. Wiegand T, Gunatilleke CVS, Gunatilleke IAUN, Hurt A. 2007 *How individual species structure diversity in tropical forests*. Proceedings of the National Academy of Science USA **104**: 19029–19033.
7. Getzin S, Wiegand T, Wiegand K, He F. 2008 Heterogeneity influences spatial patterns and demographics in forest stands. *Journal of Ecology* **96**, 807–820.
8. Wiegand T, Moloney K. 2014 *Handbook of Spatial Point-Pattern Analysis in Ecology*. Boca Raton: CRC Press.
9. Shen G, Yu M, Hu X-S, Mi X, Ren H, Sun I-F, et al. 2009 Species-area relationship explained by the joint effects of dispersal limitation and habitat heterogeneity. Ecology **90**, 3033-3041.
10. Baddeley A. 2005 Analysing spatial point patterns in R. Workshop notes. Version 4.1. CSIRO online technical publication, pp. 79-80. Available from: [www.uwa.edu.au/resources/pf16h.html](http://www.uwa.edu.au/resources/pf16h.html).
11. Baddeley A, Rubak E, Turner R. Spatial Point Patterns. 2015 *Methodology and Applications with R*. Boca Raton: CRC Press.
12. Bagchi R, Henrys PA, Brown PE, Burslem DFRP, Diggle PJ, Gunatilleke CVS et al. 2011 Spatial patterns reveal negative density dependence and habitat associations in tropical trees. *Ecology* **92(9)**, 1723-1729.
13. Shen G, He F, Waagepetersen R, Sun I-F, Hao Z-S Ch, Chen Z-S, et al. 2013 Quantifying effects of habitat heterogeneity and other clustering processes on spatial distributions of tree species. *Ecology* **94**, 2436–2443.
14. Sørbye SH, Illian JB, Simpson DP and Burslem D. Careful prior specification avoids incautious inference for log-Gaussian Cox point processes. (Submitted) [arXiv:1709.06781v1](https://arxiv.org/abs/1709.06781v1) [stat.ME].
15. Bivand RS, Pebesma E, Gómez-Rubio V. 2008 *Applied spatial data analysis with R*. 2nd ed. New York: Springer.
16. Byrd RH, Lu P, Nocedal J, Shu CA. 1995 limited memory algorithm for bound constrained optimization. SIAM J. *Scientific Computing* **16**, 1190–1208.
17. Stoyan D, Stoyan H. 1994 *Fractals, Random Shapes and Point Fields: Methods of Geometrical Statistics*. New York: John Wiley and Sons.
18. Gunatilleke CVS, Gunatilleke IAUN, Esufali S, Harms KE, Ashton PMS, Burslem DFRP, et al. 2006 Species-habitat associations in a Sri Lankan dipterocarp forest. *Journal of Tropical Ecology* **22**, 371-384.
19. Morlon H, Chuyong G, Condit R, Hubbell S, Kenfack D, Thomas D, et al. 2008 A general framework for the distance-decay of similarity in ecological communities. *Ecology Letters* **11**, 904-917.
20. Waagepetersen R. & Guan Y. 2007 Two-step estimation for inhomogeneous spatial point processes. Department of Mathematical Sciences, Aalborg University. Research Report Series, No. R-2007-25.
21. Waagepetersen R. 2008 Estimating functions for inhomogeneous spatial point process with incomplete covariate data. *Biometrika* **95**, 351-363.
22. Lin Y-Ch, Chang L-W, Yang K-Ch, Wang H-H, Sun I-F. 2011 Point patterns of tree distribution determined by habitat heterogeneity and dispersal limitation. *Oecologia* **165**, 175-184.
23. Jara-Guerrero A, De la Cruz M, Espinosa CI, Méndez M, Escudero A. 2015 Does spatial heterogeneity blur the signature of dispersal syndromes on spatial patterns of woody species? A test in a tropical dry forest. *Oikos* **124**, 1360–1366.
24. Burnham KP, Anderson DR. 2002 *Model selection and multimodel inference : a practical information-theoretic approach*. 2nd ed. New York: Springer-Verlag.
25. Webster R, McBratney AB. 1989 On the Akaike information criterion for choosing models for variograms of soil properties. *Journal of Soil Science* **40**, 493-496.
26. Webster R, Oliver MA. 2007 *Geostatistics for environmental scientists*. 2nd ed. Wiley.
